# Identification of Subunit-Subunit Interaction Sites in αA-WT Crystallin and Mutant αA-G98R Crystallin Using Isotope-Labeled Cross-Linker and Mass Spectrometry

**DOI:** 10.1371/journal.pone.0065610

**Published:** 2013-06-05

**Authors:** Rama Kannan, Puttur Santhoshkumar, Brian P. Mooney, K. Krishna Sharma

**Affiliations:** 1 Department of Biochemistry, University of Missouri, Columbia, Missouri, United States of America; 2 Department of Ophthalmology, University of Missouri, Columbia, Missouri, United States of America; 3 University of Missouri, Charles W. Gehrke Proteomics Center, Columbia, Missouri, United States of America; University of Arkansas for Medical Sciences, United States of America

## Abstract

Cataract is characterized by progressive protein aggregation and loss of vision. α-Crystallins are the major proteins in the lens responsible for maintaining transparency. They exist in the lens as highly polydisperse oligomers with variable numbers of subunits, and mutations in α-crystallin are associated with some forms of cataract in humans. Because the stability of proteins is dependent on optimal subunit interactions, the structural transformations and aggregation of mutant proteins that underlie cataract formation can be understood best by identifying the residue-specific inter- and intra-subunit interactions. Chemical crosslinking combined with mass spectrometry is increasingly used to provide structural insights into intra- and inter-protein interactions. We used isotope-labeled cross-linker in combination with LC-MS/MS to determine the subunit–subunit interaction sites in cataract-causing mutant αA-G98R crystallin. Peptides cross-linked by isotope-labeled (heavy and light forms) cross-linkers appear as doublets in mass spectra, thus facilitating the identification of cross-linker–containing peptides. In this study, we cross-linked wild-type (αA-WT) and mutant (αA-G98R) crystallins using the homobifunctional amine-reactive, isotope-labeled (d_0_ and d_4_) cross-linker–BS^2^G (bis[sulfosuccinimidyl]glutarate). Tryptic *in-solution* digest of cross-linked complexes generates a wide array of peptide mixtures. Cross-linked peptides were enriched using strong cation exchange (SCX) chromatography followed by both MS and MS/MS to identify the cross-linked sites. We identified a distinct intermolecular interaction site between K88 — K99 in the β5 strand of the mutant αA-G98R crystallin that is not found in wild-type αA-crystallin. This interaction could explain the conformational instability and aggregation nature of the mutant protein that results from incorrect folding and assembly.

## Introduction

Crystallins (α, β and γ) are the major water-soluble proteins of the lens and are responsible for its transparency. The structural interactions of crystallins and α-crystallin chaperone activity are critical to lens transparency. α-Crystallins (800–1000 kDa) constitute 40% of the lens crystallins and are made up of two subunits: αA- and αB-crystallins (20 kDa each). Chaperone activity of α-crystallin prevents the aggregation of lens proteins [1]–[3]. Loss of protein stability and α-crystallin chaperone activity causes changes in the lens architecture, leading to protein aggregation, increasing lens opacity and, ultimately, cataract development. The formation of high-molecular weight aggregates is the hallmark of cataract development, in both hereditary and age-related cataract. Hereditary, or congenital, cataract results from mutations in crystallin genes. Several αA-crystallin mutants, such as R116C, R21L, R12C, R54C and R49C, are known to cause congenital cataract [4]–[6]. G98R mutation in the αA-crystallin subunit is associated with a dominant, progressive total cataract that starts in the teenage years [7]. The mutation of G to R introduces charge and bulkiness to the α-crystallin domain of the protein to which the mutation is localized and thus disrupts the immunoglobulin fold. The mutation results in misfolded and destabilized protein, with altered secondary and tertiary structure, increased oligomeric size and a propensity to aggregate [8]–[11].

Crystallins belong to the family of small heat shock proteins, a monomer of which is characterized by a central α-crystallin domain of ∼90 amino acids, flanked by an N-terminal arm and a C-terminal extension [11], [12]. α-Crystallins assemble as polydisperse hetero-oligomers comprising a variable number of αA and αB subunits, with a constant exchange of subunits. The structural assembly of α-crystallin and therefore its chaperone function are largely regulated by appropriate interactions between the neighboring subunits [12], but identifying the subunit interaction regions by structural studies has been hampered by the large mass and polydisperse nature of the protein. The available structures of crystallins are often comprised of truncated proteins or portions removed to increase the solubility or crystallization property [13]. Still largely a mystery is the molecular basis for stability of the wild-type protein and instability and aggregation of mutant proteins in congenital cataract and the interfacial interactions that contribute to the specific characteristics of the wild-type and mutant protein.

Chemical cross-linking combined with mass spectrometry offers a promising tool for studying the structural aspects of the proteins and multiprotein complexes [14]–[16]. The technique is used to provide a topological map of multi-protein complexes and interactions sites between protein subunits as well as protein-peptide complexes. The linkage sites identified in a protein are used in structural modeling to elucidate protein conformational folds [15], [17]. Isotope -labeled cross-linkers, available as a mixture of “heavy” and “light” forms, are particularly attractive in these studies. Both forms cross-link lysine's in close proximity within and between proteins. After digestion of cross-linked species, the peptides modified by “heavy” and “light” forms carry a unique isotopic signature in mass spectra, facilitating identification of the cross-linked peptides among a large excess of non-cross-linked peptides [18], [19].

Cross-linkers are available with varied reactivities and spacer length. The most common cross-linkers are amine-reactive N-hydroxysuccinimide (NHS) esters. In this study, we used an isotope-labeled (d_0_ and d_4_) homobifunctional NHS ester, *bis*-(sulfosuccinimidyl) glutarate (BS^2^G) cross-linker, in combination with mass spectrometry to define and differentiate subunit interaction sites in native and a mutant crystallin. NHS esters form a stable amide or imide bond with the primary amines in lysine and in the N-termini of proteins, in the process releasing the NHS or sulfo-NHS group [20]. The deuterated (d_4_) and non-deuterated (d_0_) forms of the cross-linker were used in a 1∶1 ratio, and the cross-linked peptides appear as distinct doublet with a 4.025 amu mass difference in the mass spectra [21]. Using this approach we have identified a unique cross-linked site in the mutant αA-G98R-crystallin. This site is not found in wild-type protein. The location of the cross-linked site explains the conformational difference and therefore the abnormal interactions that might be responsible for aggregation of the mutant protein.

## Materials and Methods

### Materials

The cross-linking reagent Bis[Sulfosuccinimidyl] glutarate) (BS^2^G- d_0_ and d_4_) was from Proteochem, Inc (Denver, CO). Protease (Bovine) Trypsin (sequencing grade) was from G Biosciences (St Louis, MO). All other chemicals were of the highest grade commercially available.

### αA-WT and αA-G98R crystallins

Human recombinant αA-WT and αA-G98R crystallins were expressed and purified as described earlier [22]. Briefly, both recombinant proteins were expressed in *E coli* BL21(DE3) pLysS cells (Invitrogen, Carlsbad, CA). *E coli* extracts were lysed and centrifuged. Supernatants were processed for αA-WT crystallin purification, and insoluble pellets were processed for αA-G98R crystallin purification. The purification was achieved by gel-filtration (Superdex G-200) and anion-exchange chromatography (Q-Sepharose Fast Flow ion-exchange column). SDS-PAGE and mass spectrometry were used to determine the purity and molecular mass of the purified proteins.

### Cross-linking of proteins

αA-G98R-Crystallin and αA-WT crystallin (35 µM) in 50 mM phosphate buffer (pH 7.4), 150 mM NaCl (final volume 250 µl), were incubated separately at 37°C for 30 min prior to the addition of homobifunctional cross-linker BS^2^Gd_0_/d_4_ (bis[sulfosuccinimydyl]glutarate). The deuterated and non-deuterated forms of the cross-linker were prepared as 1 M stock solution in DMSO. Prior to the addition to proteins, a 1∶1 ratio of deuterated and non-deuterated form was prepared. Preliminary studies were performed using 1∶10, 1∶20, 1∶50 and 1∶100 (protein to cross-linker ratio) to determine the optimal conditions that gives good cross-linking yield. To avoid nonspecific and excessive cross-linking, a 1∶20 molar ratio was chosen and found to be optimal under the experimental conditions. A 20-fold molar excess of cross-linker mixture was added to αA-WT and αA-G98R protein separately. The samples were kept in ice for 1 hr. The cross-linking reaction was terminated by adding Tris (50 mM final concentration). After incubation at room temperature for 10 min, the samples were passed through 10 kDa centrifugal filters (Centricon) to remove unreacted cross-linker. Aliquots of the reaction mixture were run on 4–20% SDS-PAGE.

### In-solution digestion of cross-linked proteins

In-solution digestion was carried out with a modified procedure described earlier [23]. In order to make the proteolytic sites more accessible to trypsin, the cross-linked protein samples (αA-WT and αA-G98R) were solubilized in 50 mM Tris buffer containing 8 M urea and 4 mM DTT for 2 h and then 12.5-fold diluted (final urea and DTT concentration of 0.64 M and 0.32 mM respectively) by adding trypsin digestion buffer (0.2 M ammonium bicarbonate, pH 7.9). Trypsin (1.75 µg) (sequencing grade Trypsin, G. Biosciences) was added to 175 µg of cross-linked proteins. The trypsin-treated mixtures were incubated overnight at 37°C.

### Enrichment of cross-linked peptides

The tryptic digests of cross-linked αA-WT and αA-G98R protein were off-line fractionated using SCX-Stage tips (Thermo Scientific), following the manufacturer's protocol. Peptides were eluted from the SCX column using a stepwise gradient of ammonium acetate. Eluted fractions were pooled and used for LC-MS analysis.

### Mass spectrometry analysis

SCX-enriched peptide mixtures (1 µl) were separated on an Agilent HPLC chip (43 mm, Zorbax C18 Chip) and directly coupled to Agilent 6520 Accurate-Mass Quadrupole time-of-flight (Q-TOF) LC/MS. Elution was done using gradients with initial conditions: 3% B to 10% B over 1 min, 10% B to 40% B over 22.5 min, 40% B to 90% B over 1 min, hold at 90% B for 5 min and back to initial conditions at 3% B for 4 min, with a total run time of 35.5 min. Solvent A is 0.1% formic acid in water, solvent B is 99.9% acetonitrile and 0.1% formic acid. Flow rate was maintained at 600 nL/min. MS spectra of the eluting peptides were acquired in the range of 295–2500 m/z, 2 spectra/sec. For each cycle of MS scan (3.1 sec), the five most abundant peptides (>2500 counts) with a charge state of two, three or higher were selected and subjected to N2-induced CID peptide fragmentation (MSMS, 7–2500 m/z, 2 spectra/sec). Two internal reference mass compounds (methyl stearate 299.29 and hexasis [1H, 1H, 4H-hexafluorobutyloxy] phosphazine 1221.99) were used to recalibrate mass spectra during acquisition.

### Identification of cross-linked peptides

LC-MS raw data were processed into deconvoluted peptide peak lists with monoisotopic mass using “find compounds by molecular feature” algorithm of Mass Hunter Qualitative Analysis Software B.02.00. Theoretical mass lists of the cross-linked peptides were generated using GPMAW (General Protein Mass Analysis for Windows) version 9.20 (Lighthouse Data, Odense, Denmark). Search parameters were enzyme, Trypsin; crosslinker, BS^2^G; maximum missed cleavages, two; cross-linkable amino acids, N-terminus and lysine; mass limit, 8000; and no cleavage at modified residues. The *in-silico*-generated mass lists were compared with the mass lists generated by mass spectrometry with a mass tolerance of 10 ppm. Potential cross-linked peptide ions were chosen for fragmentation by tandem MS/MS. Interpretation of the MS/MS spectra was done manually following *de novo* sequencing.

## Results

### Cross-linking of proteins

**Figure 1:**
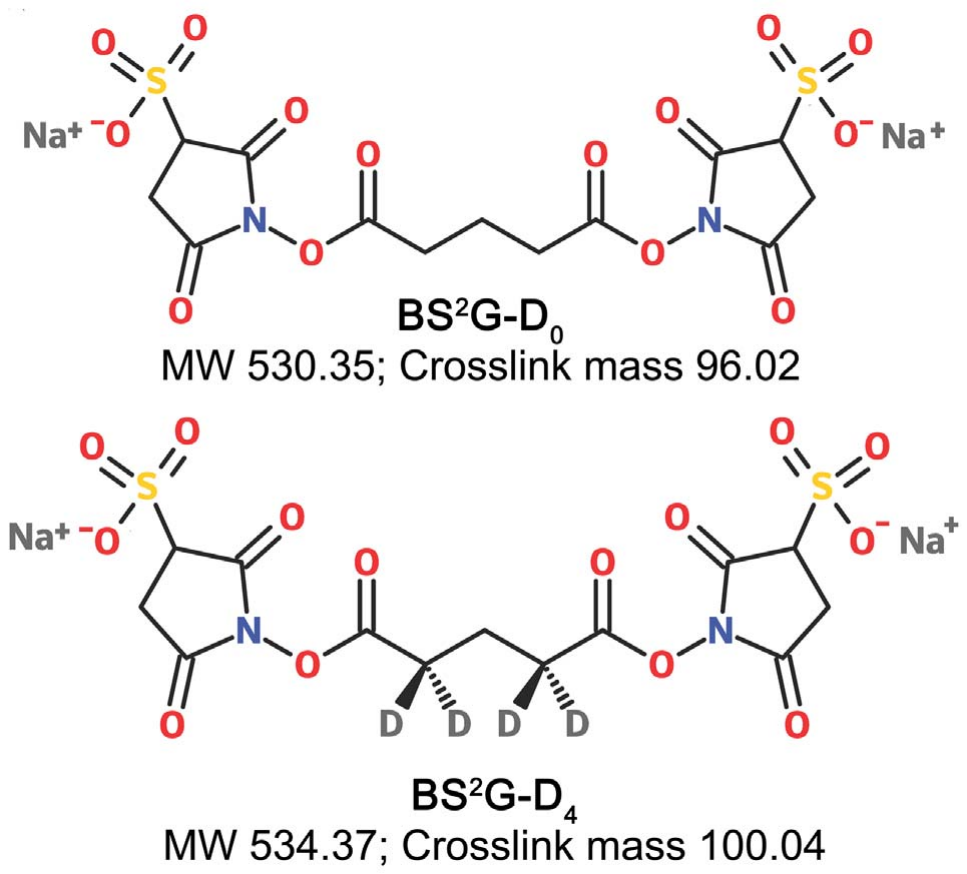
Amine-reactive NHS ester cross-linker BS^2^G- (Bis[sulfosuccinimidyl] glutarate) in its light and heavy form. Four hydrogen atoms in light (d_0_) form are replaced by four deuterium atoms in heavy (d_4_) form. The corresponding mass shifts associated with d_0_ and d_4_ cross-linked peptides are shown.

**Figure 2:**
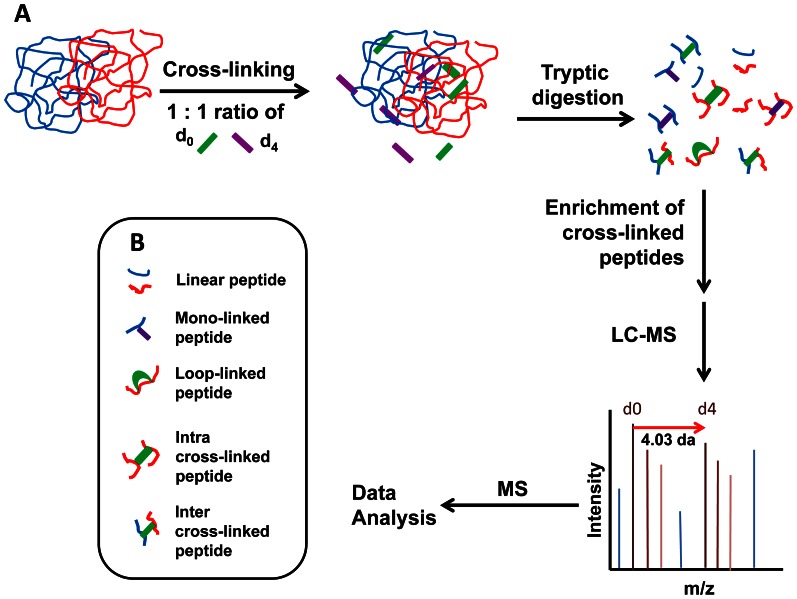
Identification of the interaction sites in protein complexes by isotope-labeled cross-linking and mass spectrometry. A— Strategy for cross-linking. The interacting partners are treated with equimolar amounts of light and heavy cross-linkers. Following labeling the samples are digested, enriched and analyzed by LC-MS. Inter cross-linked peptides were identified by GPMAW software and confirmed by MS/MS analysis. B— The peptide types generated after digestion of the cross-linked proteins.

**Figure 3:**
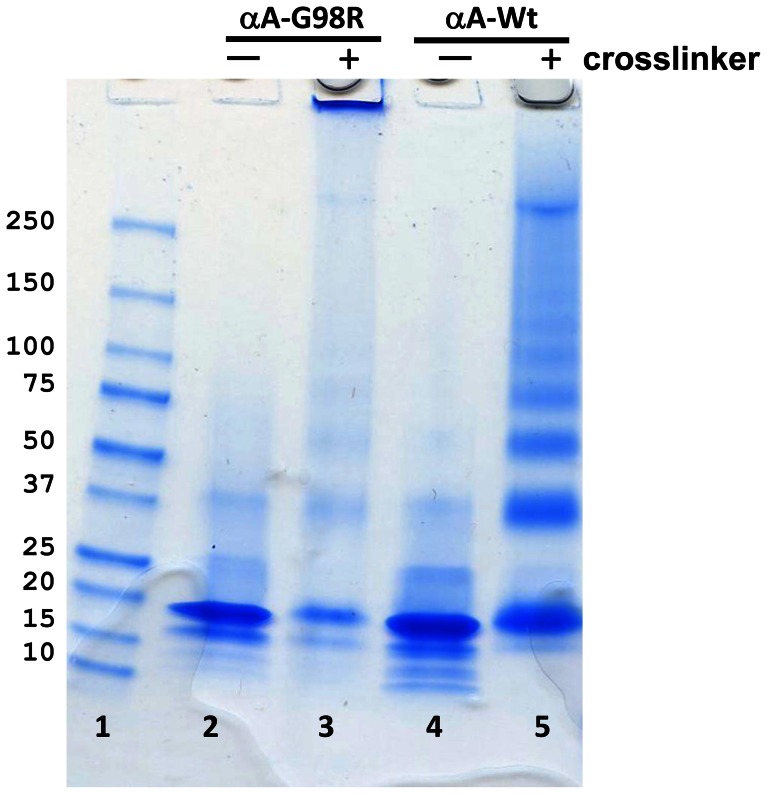
SDS-PAGE analysis of cross-linked protein products. αA-WT crystallin and αA-G98R crystallin are preincubated at 37°C, as indicated under methods, and cross-linked with 20-fold molar excess of BS ^2^G d_0_/d_4_, for 60 min in ice. Aliquots of the samples are separated on a 4–20% acrylamide SDS-PAGE and stained with Coomassie blue. Molecular weight marker (lane1), non-crosslinked control αA- G98R (lane 2), BS^2^G d_0_/d_4_ crosslinked αA-G98R (lane 3), non-crosslinked control αA-WT (lane 4), BS^2^G d_0_/d_4_ crosslinked αA –WT (lane 5). Crosslinked αA-WT sample (lane 5) shows cross-linked species as dimers, trimers and oligomers, whereas the cross-linked αA-G98R sample (lane3) under the same experimental conditions forms higher-order complexes that did not enter the gel.

To identify the subunit–subunit interaction sites in αA-WT and αA-G98R crystallins, deuterated and non-deuterated form of a homobifunctional cross-linker BS^2^G were used to cross-link the proteins (Figure 1). BS^2^G reacts with protein N-terminal α-amino and lysine ε-amino groups. The analytical strategy used to characterize the intra- and inter-subunit interaction sites in αA-WT and αA-G98R crystallin is illustrated in Figure 2. To identify the initial inter-subunit interactions that initiate aggregation, we pre-incubated the αA-WT and G98R proteins at 37°C for 30 min, before adding the cross-linker. Mild thermal stress at 37°C induces the process of aggregation in the mutant G98R protein [9], [10]. After incubation, cross-linker was added in 10-, 20-, 50- and 100-fold molar excess to determine the optimal cross-linker to protein ratio. The reaction mixtures were kept in ice for 1 hr to suppress subunit exchange during crosslinking. After termination of crosslinking reactions and removal of unreacted cross-linker by filtration, an aliquot of the sample was boiled and run in SDS-PAGE (Figure 3). We did not observe sufficient cross-linking in samples containing 1∶10 protein to cross-linker ratio (data not shown). At proteins to crosslinker ratios of 1∶20 and above, cross-linking of αA-WT crystallin was characterized by the appearance of ladder of bands corresponding to dimer, trimer, tetramer, etc. (Figure 3, Lane 5 and Figure S1A). With αA-WT, the extent of cross-linking and the gel pattern appeared the same at all protein to cross-linker ratios and at all-time points of cross-linking reaction tested. G98R crystallin, being an aggregation-prone mutant protein, formed high mass aggregates visible on top of the gel at all protein to cross-linker ratios tested (Figure 3, Lane 3 and Fig. S1B). For further analysis, a molar ratio of 1∶20 was chosen in order to minimize nonspecific cross-links.

The cross-linked samples were digested in solution with trypsin prior to reversed-phase LC separation and MS/MS analysis. Trypsin cleavage results in peptides with a basic residue at the carboxy terminus, and if the basic lysine residue is cross-linked, it results in missed cleavage [24]. Protease digestion results in complex mixtures of mono-linked, loop-linked and interlinked peptides (Figure 2B). Among these the desired cross-linked product will be present in low abundance amidst the large excess of unwanted, unmodified peptide [19]. Strong cation exchange (SCX) chromatography has been successfully used to enrich the cross-linked peptides [25]–[27]. SCX chromatography is based on the charge difference between the cross-linked and non-cross-linked peptides. Cross-linked peptides have a higher charged state than non-cross-linked peptides. Thus, the cross-linked peptides can be eluted from SCX chromatography using high salt concentrations [19], [26], [28]. Therefore, in our experiments we enriched the targeted cross-linked peptides using SCX-Stage tips. LC-MS analysis of enriched peptide mixtures from SCX chromatography was done and the mono-isotopic deconvoluted m/z values of 500 high intensity peaks were selected for comparison with theoretical mass lists generated by GPMAW software. Lysines in the proteins were chosen as reaction sites of the cross-linker in GPMAW. The cross-linking reagent and the potential amino acids in the proteins that can be cross-linked were defined. αA-WT has seven lysines, which can potentially be modified by the cross-linker.

**Figure 4:**
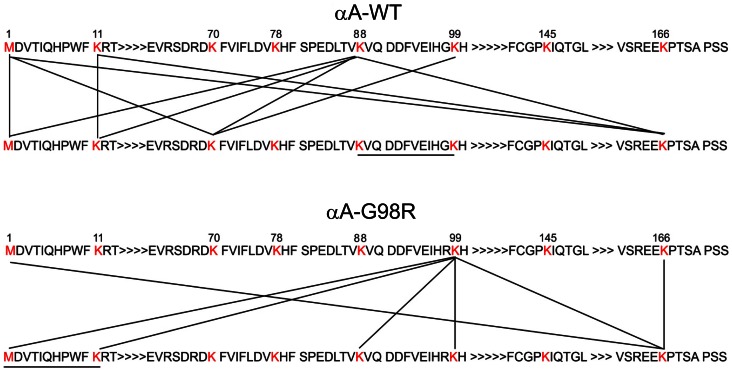
Map of potential cross-linked sites in αA-WT (top) and αA-G98R (bottom). Lysine and N-termini cross-links within and between subunits in proteins are shown as connecting lines. Theoretical mass lists of the cross-linked peptides are generated using GPMAW software. In GPMAW, lysines in αA-WT and αA-G98R crystallin are selected and tryptic cleavage products are generated. Since trypsin will not cleave at modified lysines (involved in cross-links), two missed cleavage sites is set as a parameter. A m/z mass difference of 4 amu is set as a criterion to create mass lists corresponding to cross-linked peptide pairs. The mass lists generated by GPMAW are compared with experimental mass lists obtained by LC-MS analysis of tryptic digests of cross-linked αA-WT and αA-G98R (Table 1). Based on the match, a list of potential cross-linked peptides is created. Sequences of the corresponding cross-linked sites in proteins are identified in-silico and represented as a map. The links in the map represent cross-link sites within and between subunits in αA-WT and αA-G98R crystallins, respectively (Table 1).

**Figure 5:**
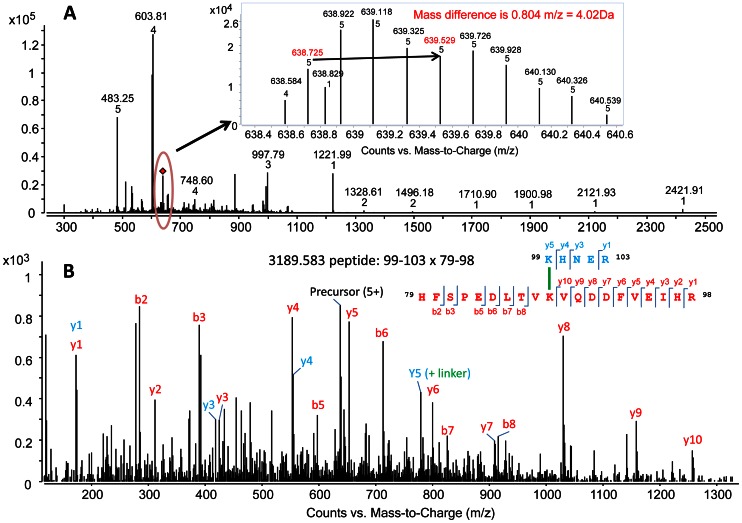
LC-MS and MS/MS analysis of a cross-linked αA-G98R peptide. A— ESI mass spectrum of peptides from tryptic digest of αA-G98R crystallin cross-linked with 20-fold molar excess of BS^2^G d_0_/d_4_, eluting at 9.7 min in HPLC. The signal region circled in the chromatogram is expanded to show the cross-linked peptide pairs (light and heavy, (m/z 638.7 for d_0_ species and m/z 639.5 for d_4_ species) having a charge state of +5 and differing by a mass (m/z) of 0.804. (Please note that 638.7 m/z is the +5 charged species whose neutral monoisotopic (+1) mass is 3189.57 (Highlighted in Table 1). The signal highlighted in the inset was analyzed by MS/MS. B— Product ion mass spectrum of +5 charged precursor (m/z 638.7 for d_0_ species) of the inter-protein cross-linked αA-G98R crystallin. The spectrum represents two separate peptides from αA-G98R comprising amino acids 99–103 and 79–98 cross linked at Lys88 and Lys99. The spectrum exhibits peaks corresponding to b and y ion series from fragmentation of each peptide. Peptide sequences with cross-linked site and the identified fragment ions are indicated in the upper right. The b and y ions are labeled in the spectra and indicated on the peptide sequence in upper right) MS/MS data were collected in the m/z range 70–2500 Da. Larger fragment ions are not observed as the MS/MS signal significantly drops above 2000 Da.

Peptides cross-linked with heavy forms (d_4_) appear with a characteristic mass shift of 100.045 amu, and peptides cross-linked with light forms (d_0_) show a mass shift of 96 amu. Thus the cross-linked peptides appear as doublet mass signals with a mass difference of 4.025 Da, 2.0123 Da and 1.341 Da for mono, double and triple protonated forms, thus facilitating identification by mass spectrometry. BS^2^G- d_0_ and d_4_ cross-linked ion pairs with the same retention time and a delta m/z of 4.0247 were used as filters to identify and generate the list of peptide doublets in LC-MS data after comparison with GPMAW data (Table 1). A map of linkage sites between subunits in αA-WT crystallin and αA-G98R crystallin was developed based on the match (Figure 4). In αA-WT crystallin, the major site of cross-linking is at K88, and only one linking site is seen at K99. In contrast, in mutant αA-G98R crystallin, the major interaction point shifts to K99, and only one linking site is seen at K88. This difference in linkage sites is significant as the structural arrangement and conformation of WT and mutant crystallins are different [10], [29], and therefore different cross-linked products are expected. Cross-linked peptide ion pairs were subjected to MS/MS for identification of cross-linked sites. Except for one, other cross-linked peptides identified in MS were not amenable to MS/MS analysis due to low abundance and changes in their ionization potential [19]. Figure 5A shows the MS/MS fragmentation spectra of a distinct cross-linked peptide observed in mutant G98R protein, but not in αA-WT protein. At the elution time point of 9.7 min, the average mass spectrum of the mutant G98R crystallin exhibit the signal of an inter-subunit cross-linked product at 3189.58 [MH^+^]. MS/MS analysis of the precursor ion shows the cross-link between K99 of one G98R subunit and K88 of another G98R subunit (Figure 5B). This interaction site is attributed to a conformational rearrangement within the mutant protein due to replacement of neutral glycine to positively charged arginine.

**Table 1:** αA-WT and αA-G98R crystallin cross-links identified based on increase in mass corresponding to peptides cross-linked with deuterated and non-deuterated reagents.

	Experimental data		GPMAW analysis				
d_0_/d_4_ Pair	RT(min)	(MH+)^a^	(MH+)^b^	αA-wt ^c^	αA-wt ^d^	Type ^e^	ppm ^f^
1	5.593	3312.705	3312.705	158–173	1–12	X-link	0
	5.598	3308.684	3308.673	158–173	1–12	X-link	−3
2	9.256	4195.113	4195.119	158–173	79–99	X-link	2
	9.261	4191.090	4191.088	158–173	79–99	X-link	0
3	11.831	3076.437	3076.512	79–103		Int. X-link	1
	11.843	3072.416	3072.481	79–103		Int. X-link	−2
4	13.796	4097.042	4097.059	79–99	1–12	X-link	4
	13.797	4093.026	4093.028	79–99	1–12	X-link	0
5	14.541	4657.343	4657.369	89–103	66–88	X-link	5
	14.546	4653.330	4653.337	89–103	66–88	X-link	2
			4657.369	79–103	66–78	X-link	5
			4653.337	79–103	66–78	X-link	2
6	8.773	3312.690	3312.705	158–173	1–12	X-link	4
	8.777	3308.675	3308.673	158–173	1–12	X-link	−1
7	11.461	3214.629	3214.644	1–12	1–12	X-link	5
	11.466	3210.611	3210.613	1–12	1–12	X-link	1
8	15.025	3238.703	3238.708	66–78	1–12	X-link	2
	15.031	3234.674	3234.677	66–78	1–12	X-link	1

d_0_/d_4_ Pair	RT (min)	(MH+)^a^	(MH+)^b^	αA-G98R ^c^	αA-G98R ^d^	Type ^e^	ppm ^f^
1	2.684	2434.249	2434.232	158–173	99–103	X-link	−7
	2.696	2438.255	2438.263	158–173	99–103	X-link	3
2	4.475	3410.763	3410.765	158–173	158–173	X-link	1
	4.492	3406.741	3406.734	158–173	158–173	X-link	−2
**3** *	**9.759**	**3189.587**	**3189.571**	**99–103**	**79–98**	**X-link**	**−3**
	**9.759**	**3193.614**	**3193.603**	**99–103**	**79–98**	**X-link**	**−4**
4	9.987	1657.847	1657.852	1–12		Int. X-link	3
	9.993	1653.824	1653.821	1–12		Int. X-link	−2
5	2.010	2537.243	2537.235	99–103	99–112	X-link	−3
	2.028	2533.227	2533.204	99–103	99–112	X-link	−9
6	2.450	3608.710	3608.709	99–112	99–112	X-link	0
	2.452	3604.685	3604.677	99–112	99–112	X-link	−2
7	2.709	3505.724	3505.705	158–173	99–112	X-link	−5
	2.736	3509.750	3509.737	158–173	99–112	X-link	−4
8	3.078	3509.732	3509.737	158–173	99–112	X-link	1
	3.079	3505.714	3505.705	158–173	99–112	X-link	−2
9	5.959	2340.203	2340.203	99–103	1–12	X-link	0
	5.961	2336.184	2336.172	99–103	1–12	X-link	−5
10	8.864	3312.699	3312.705	158–173	1–12	X-link	2
	8.864	3308.682	3308.673	158–173	1–12	X-link	−3

The monoisotopic masses of the GPMAW identified peptide pairs (experimental^a^, theoretical^b^ and the calculated mass difference between the two in ppm^f^) are given. Subunit-subunit interaction sequences^c, d^ in αA-WT and αA-G98R are given. The crosslink type is indicated as X-link (between the subunits) and Int-X-link (within the subunits)^e^.

* Sequence confirmed by MS/MS analysis.

Ion pairs having similar retention time (RT) in LC and differing by mass of 4.03 Da were generated from the MS data using Excel spreadsheet. The theoretical m/z values for the cross-linked dipeptides were obtained from the software GPMAW (General Protein/Mass Analysis for Windows, Version 9.21, Lighthouse data; (http://www.gpmaw.com), using the MS X-link function for the respective protein and cross-linker and allowing two missed cleavage sites for trypsin. The experimental m/z values were searched against the theoretical m/z values using the Mass search feature of GPMAW set at 10 ppm.

## Discussion

G98R-mutated αA-crystallin exhibits structural and functional differences from αA-WT crystallins [9], [10]. These include secondary and tertiary structural perturbations resulting in larger oligomeric size, decreased stability, altered chaperone ability and folding defects in the mutant protein, as reported earlier [9], [10]. Increased aggregation propensity of the mutant protein underlies the molecular basis for the lens turbidity and cataract formation. Subunit interactions determine the self-assembly and organization of native proteins. Therefore, it is imperative that these interactions be altered in order for mutant proteins to have increased oligomeric size and aggregation propensity. [9], [30], [31]. We hypothesized that determining the subunit interaction sites in αA-WT crystallin and αA-G98R crystallin would provide key insights into the molecular basis for the structural conversions leading to the crystallin aggregation. We used chemical cross linking combined with mass spectrometry to identify the subunit interaction regions in αA-WT and αA-G98R crystallins. Such an approach has been successfully used to identify subunit-subunit interactions in native α-crystallin [32], [33], phage proteins [34], quaternary domain interactions in Hsp90 chaperones [35], interaction sites in soluble aggregates of monoclonal antibody [36] and sHSP21 and substrate interactions [37], etc.

We report hitherto unreported inter-subunit crosslinks in α-crystallin domain of αA-WT and G98R crystallins. Our cross-linking studies reveal that majority of the inter-subunit cross-linking is clustered in the K88 region in αA-WT and in the K99 region in mutant αA-G98R protein (Figure 4). K99 in αA-WT crystallin is solvent exposed and is not proximal to any other amino group, as has been shown using DTSSP cross-linker [32], [33]. In agreement with these studies, we observed no inter-subunit interactions involving K99 in αA-WT, but did note inter-subunit interactions in the K99 region in mutant G98R protein. The β-sandwich assembly comprising β2–β9 strands in the α-crystallin domain [38] forms an anti-parallel (AP) interface comprising the dimer, which forms the basic assembly unit for the higher order oligomerization of the wild-type protein [39]–[41]. The oligomerization involves the formation of various inter-chain interactions involving ion pairs between topologically equivalent residues [8], [36]. G98R mutation in αA-crystallin introduces a charged amino acid, which could have resulted in the gain of ion pairs in the interface not seen in WT proteins. The cross-link observed in G98R mutant proteins between K88–K99 in the β5 strand could have resulted from the close proximity of the two lysines as a result of a conformational change different from the WT protein. Such new and altered interfacial interactions in G98R could have affected the subunit exchange dynamics and the structural organizations required for protein stability and lens transparency. Our findings support the earlier view that mutant proteins have increased and different interfacial interactions not found in wild-type proteins [11], [29], [42] and has uncovered the specific changes in α-crystallin domain. Further analysis of subunit arrangements in proteins could help in constructing a structural model and therefore the long-range consequences of such mutations.

In conclusion, the results reveal a new, previously unknown interaction site between G98R subunits. The difference in the cross-linking pattern between the αA-WT and G98R crystallin likely reflects the different oligomerization of the proteins due to altered subunit interaction regions. Our studies demonstrate the use of chemical cross-linking and mass spectrometry as a tool for expanding our understanding of the interactions and conformational changes in mutant proteins that contribute to their aggregation.

## Supporting Information

Figure S1 **SDS-PAGE of BS^2^Gd_0_ cross-linked αA-WT and G98R crystallin (50- and 100-fold molar excess of cross-linker).** Cross-linking reactions contained 25 µM of protein in 50 mM Phosphate buffer (pH 7.4) (final volume 500 µl). The reactions were carried out in ice for 2 hr, and 100 µl aliquots were drawn at 5, 15, 30, 60 and 120 min. The reactions were terminated by adding Tris (final concentration 50 mM) to each aliquot. A—SDS-PAGE of cross-linked αA-WT crystallin - 1∶50 and 1∶100 at different time points. B—SDS-PAGE of cross-linked G98R crystallin- 1∶50 and 1∶100 at different time points. Although the cross-linking occurs in both WT and G98R αA-crystallin in 5 min the profiles are distinct on SDS-PAGE.
(TIF)

## References

[1] Horwitz J (1992) α-Crystallin can function as a molecular chaperone. Proc Natl Acad Sci USA 89: 10449–10453. 1438232 10.1073/pnas.89.21.10449 PMC50356

[2] Wang K, Spector A (1994) The chaperone activity of bovine α crystallin. Interaction with other lens crystallins in native and denatured states. J Biol Chem 269: 13601–13608. 7909809

[3] Rao PV, Huang QL, Horwitz J, Zigler JS Jr (1995) Evidence that α-crystallin prevents non-specific protein aggregation in the intact eye lens. Biochim Biophys Acta 1245: 439–447. 8541324 10.1016/0304-4165(95)00125-5

[4] Litt M, Kramer P, LaMorticella DM, Murphey W, Lovrien EW, et al (1998) Autosomal dominant congenital cataract associated with a missense mutation in the human alpha crystallin gene CRYAA. Hum Mol Genet 7: 471–474. 9467006 10.1093/hmg/7.3.471

[5] Graw J, Klopp N, Illig T, Preising MN, Lorenz B (2006) Congenital cataract and macular hypoplasia in humans associated with a de novo mutation in CRYAA and compound heterozygous mutations in P. Graefes Arch Clin Exp Ophthalmol 244: 912–919. 16453125 10.1007/s00417-005-0234-x

[6] Graw J (2009) Genetics of crystallins: Cataract and beyond. Exp Eye Res 88: 173–189. 19007775 10.1016/j.exer.2008.10.011

[7] Santhiya ST, Söker T, Klopp N, Illig T, Prakash MVS, et al (2006) Identification of a novel, putative cataract-causing allele in CRYAA (G98R) in an Indian family. Mol Vis 12: 768–773. 16862070

[8] Mornon JP, Halaby D, Malfois M, Durand P, Callebaut I, et al (1998) α-crystallin C-terminal domain: On the track of an Ig fold. Int J Biol Macromol 22: 219–227. 9650076 10.1016/s0141-8130(98)00019-1

[9] Singh D, Raman B, Ramakrishna T, Rao CM (2006) The cataract-causing mutation G98R in human αA-crystallin leads to folding defects and loss of chaperone activity. Mol Vis 12: 1372–1379. 17149363

[10] Murugesan R, Santhoshkumar P, Sharma KK (2007) Cataract-causing αAG98R mutant shows substrate-dependent chaperone activity. Mol Vis 13: 2301–2309. 18199971

[11] Clark AR, Lubsen NH, Slingsby C (2012) SHSP in the eye lens: Crystallin mutations, cataract and proteostasis. Int J Biochem Cell Biol 44: 1687–1697. 22405853 10.1016/j.biocel.2012.02.015

[12] Bloemendal H, De Jong W, Jaenicke R, Lubsen NH, Slingsby C, et al (2004) Ageing and vision: Structure, stability and function of lens crystallins. Prog Biophys Mol Bio 86: 407–485. 15302206 10.1016/j.pbiomolbio.2003.11.012

[13] Horwitz J (2009) Alpha crystallin: The quest for a homogeneous quaternary structure. Exp Eye Res 88: 190–194. 18703051 10.1016/j.exer.2008.07.007 PMC2678943

[14] Young MM, Tang N, Hempel JC, Oshiro CM, Taylor EW, et al (2000) High throughput protein fold identification by using experimental constraints derived from intramolecular cross-links and mass spectrometry. Proc Natl Acad Sci USA 97: 5802–5806. 10811876 10.1073/pnas.090099097 PMC18514

[15] Sinz A (2006) Chemical cross-linking and mass spectrometry to map three-dimensional protein structures and protein-protein interactions. Mass Spectrom Rev 25: 663–682. 16477643 10.1002/mas.20082

[16] Sinz A (2007) Isotope-labeled photoaffinity reagents and mass spectrometry to identify protein-ligand interactions. Angew Chem Int Edit 46: 660–662. 10.1002/anie.200602549 17167803

[17] Rappsilber J (2011) The beginning of a beautiful friendship: Cross-linking/mass spectrometry and modelling of proteins and multi-protein complexes. J Struct Biol 173: 530–540. 21029779 10.1016/j.jsb.2010.10.014 PMC3043253

[18] Müller DR, Schindler P, Towbin H, Wirth U, Voshol H, et al (2001) Isotope-tagged cross-linking reagents. A new tool in mass spectrometric protein interaction analysis. Anal Chem 73: 1927–1934. 11354472 10.1021/ac001379a

[19] Leitner A, Walzthoeni T, Kahraman A, Herzog F, Rinner O, et al (2010) Probing native protein structures by chemical cross-linking, mass spectrometry, and bioinformatics. Mol Cell Proteomics 9: 1634–1649. 20360032 10.1074/mcp.R000001-MCP201 PMC2938055

[20] Kalkhof S, Sinz A (2008) Chances and pitfalls of chemical cross-linking with amine-reactive N-hydroxysuccinimide esters. Anal Bioanal Chem 392: 1–8. 18724398 10.1007/s00216-008-2231-5

[21] Ihling C, Schmidt A, Kalkhof S, Schulz DM, Stingl C, et al (2006) Isotope-Labeled Cross-Linkers and Fourier Transform Ion Cyclotron Resonance Mass Spectrometry for Structural Analysis of a Protein/Peptide Complex. J Am Soc Mass Spectrom 17: 1100–1113. 16750914 10.1016/j.jasms.2006.04.020

[22] Raju M, Santhoshkumar P, Krishna Sharma K (2011) Cataract-causing αAG98R-crystallin mutant dissociates into monomers having chaperone activity. Mol Vis 17: 7–15. 21224997 PMC3017799

[23] Du J, Murphy RM (2010) Characterization of the interaction of beta-amyloid with transthyretin monomers and tetramers. Biochemistry 49: 8276–8289. 20795734 10.1021/bi101280t PMC2943652

[24] Steen H, Mann M (2004) The ABC's (and XYZ's) of peptide sequencing. Nat Rev Mol Cell Bio 5: 699–711. 15340378 10.1038/nrm1468

[25] Maiolica A, Cittaro D, Borsotti D, Sennels L, Ciferri C, et al (2007) Structural analysis of multiprotein complexes by cross-linking, mass spectrometry, and database searching. Mol Cell Proteomics 6: 2200–2211. 17921176 10.1074/mcp.M700274-MCP200

[26] Rinner O, Seebacher J, Walzthoeni T, Mueller L, Beck M, et al (2008) Identification of cross-linked peptides from large sequence databases. Nat Met 5: 315–318. 10.1038/nmeth.1192 PMC2719781 18327264

[27] Chen ZA, Jawhari A, Fischer L, Buchen C, Tahir S, et al (2010) Architecture of the RNA polymerase II-TFIIF complex revealed by cross-linking and mass spectrometry. EMBO J 29: 717–726. 20094031 10.1038/emboj.2009.401 PMC2810376

[28] Fritzsche R, Ihling CH, Götze M, Sinz A (2012) Optimizing the enrichment of cross-linked products for mass spectrometric protein analysis. Rapid Commun Mass Sp 26: 653–658. 10.1002/rcm.6150 22328219

[29] Kore R, Hedges RA, Oonthonpan L, Santhoshkumar P, Sharma KK, et al (2012) Quaternary structural parameters of the congenital cataract causing mutants of αa-crystallin. Mol Cell Biochem 361: 93–102. 10.1007/s11010-011-1131-8 PMC3788686 22045060

[30] Jaenicke R, Seckler R (1997) Protein misassembly in vitro. Adv Protein Chem pp 1–59. 10.1016/s0065-3233(08)60318-6 9338078

[31] Singh D, Raman B, Ramakrishna T, Rao CM (2007) Mixed Oligomer Formation between Human αA-Crystallin and its Cataract-causing G98R Mutant: Structural, Stability and Functional Differences. J Mol Biol 373: 1293–1304. 17900621 10.1016/j.jmb.2007.08.062

[32] Peterson JJ, Young MM, Takemoto LJ (2004) Probing α-crystallin structure using chemical cross-linkers and mass spectrometry. Molecular Vision 10: 857–866. 15570221

[33] Swaim CL, Smith DL, Smith JB (2004) The reaction of α-crystallin with the cross-linker 3,3′-dithiobis(sulfosuccinimidyl propionate) demonstrates close proximity of the C termini of αA and αB in the native assembly. Protein Science 13: 2832–2835. 15388868 10.1110/ps.04910004 PMC2286569

[34] Kang S, Hawkridge AM, Johnson KL, Muddiman DC, Prevelige PE Jr (2006) Identification of subunit-subunit interactions in bacteriophage P22 procapsids by chemical cross-linking and mass spectrometry. J Proteome Res 5: 370–377. 16457603 10.1021/pr050356f

[35] Chu F, Maynard JC, Chiosis G, Nicchitta CV, Burlingame AL (2006) Identification of novel quaternary domain interactions in the Hsp90 chaperone, GRP94. Protein Sci 15: 1260–1269. 16731965 10.1110/ps.052065106 PMC2242539

[36] Zhao A, Hao G, Gu J (2013) Chemical crosslinking and mass spectrometric identification of interaction sites within soluble aggregate of protein therapeutics. Journal of Pharmaceutical and Biomedical Analysis 73: 99–102. 22677652 10.1016/j.jpba.2012.05.006

[37] Lambert W, Rutsdottir G, Hussein R, Bernfur K, Kjellstrom S, et al (2013) Probing the transient interaction between the small heat-shock protein Hsp21 and a model substrate protein using crosslinking mass spectrometry. Cell Stress Chaperones 18: 75–85. 22851138 10.1007/s12192-012-0360-4 PMC3508123

[38] Van Montfort RLM, Basha E, Friedrich KL, Slingsby C, Vierling E (2001) Crystal structure and assembly of a eukaryotic small heat shock protein. Nature Structural Biology 8: 1025–1030. 11702068 10.1038/nsb722

[39] Bagneris C, Bateman OA, Naylor CE, Cronin N, Boelens WC, et al (2009) Crystal structures of alpha-crystallin domain dimers of alphaB-crystallin and Hsp20. J Mol Biol 392: 1242–1252. 19646995 10.1016/j.jmb.2009.07.069

[40] Laganowsky A, Benesch JL, Landau M, Ding L, Sawaya MR, et al (2010) Crystal structures of truncated alphaA and alphaB crystallins reveal structural mechanisms of polydispersity important for eye lens function. Protein Sci 19: 1031–1043. 20440841 10.1002/pro.380 PMC2868245

[41] Baranova EV, Weeks SD, Beelen S, Bukach OV, Gusev NB, et al (2011) Three-dimensional structure of alpha-crystallin domain dimers of human small heat shock proteins HSPB1 and HSPB6. J Mol Biol 411: 110–122. 21641913 10.1016/j.jmb.2011.05.024

[42] Clark AR, Naylor CE, Bagnéris C, Keep NH, Slingsby C (2011) Crystal structure of R120G disease mutant of human αb-crystallin domain dimer shows closure of a groove. J Mol Biol 408: 118–134. 21329698 10.1016/j.jmb.2011.02.020 PMC3158665

