# Supplementary figures and images for: Identification of Subunit-Subunit Interaction Sites in αA-WT Crystallin and Mutant αA-G98R Crystallin Using Isotope-Labeled Cross-Linker and Mass Spectrometry
Source: PLoS One. 2013 Jun 5;8(6):e65610. doi: 10.1371/journal.pone.0065610 (PMC3673982; doi:10.1371/journal.pone.0065610)

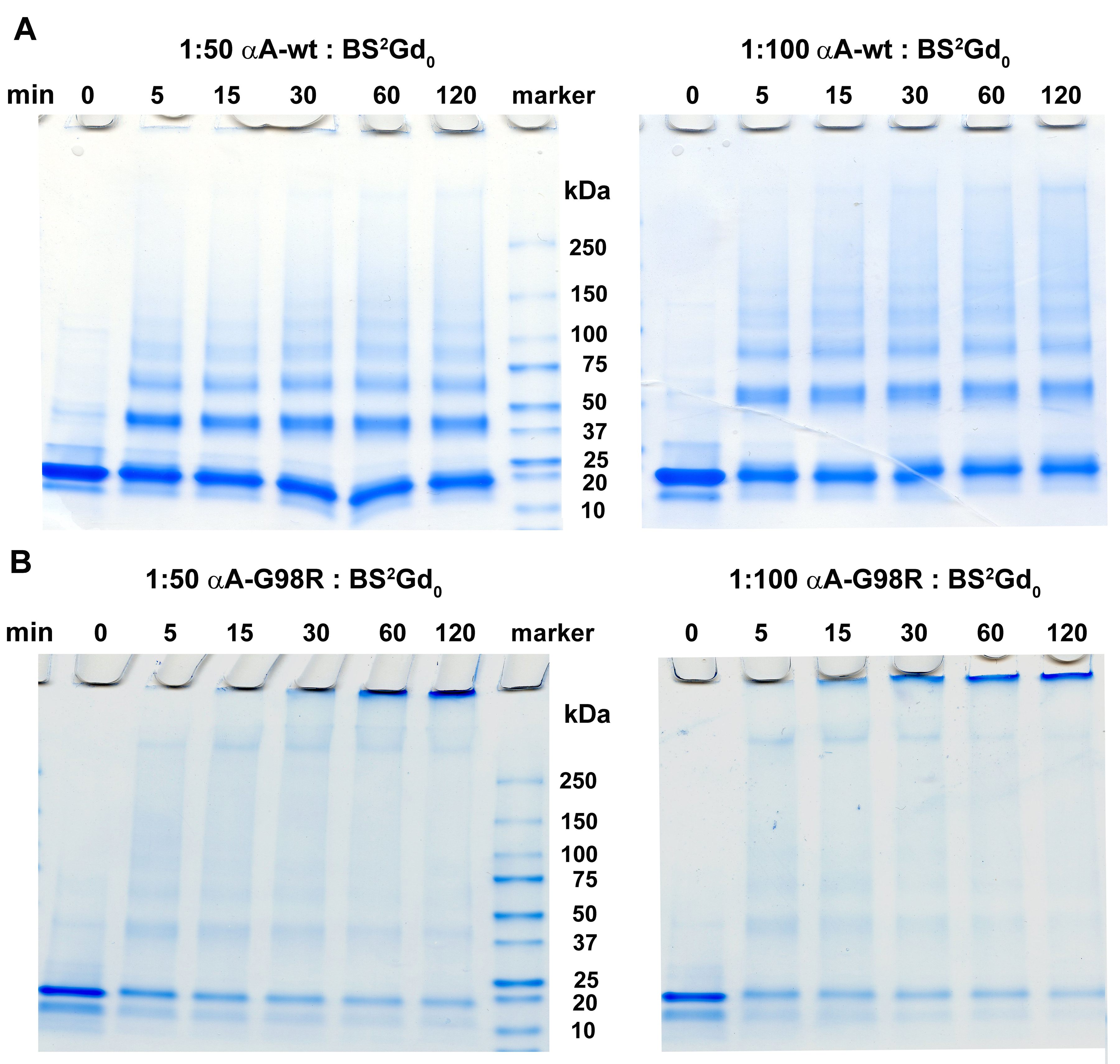

Supplement: Figure S1 — SDS-PAGE of BS2Gd0 cross-linked αA-WT and G98R crystallin (50- and 100-fold molar excess of cross-linker). Cross-linking reactions contained 25 µM of protein in 50 mM Phosphate buffer (pH 7.4) (final volume 500 µl). The reactions were carried out in ice for 2 hr, and 100 µl aliquots were drawn at 5, 15, 30, 60 and 120 min. The reactions were terminated by adding Tris (final concentration 50 mM) to each aliquot. A—SDS-PAGE of cross-linked αA-WT crystallin - 1∶50 and 1∶100 at different time points. B—SDS-PAGE of cross-linked G98R crystallin- 1∶50 and 1∶100 at different time points. Although the cross-linking occurs in both WT and G98R αA-crystallin in 5 min the profiles are distinct on SDS-PAGE. (TIF) [file pone.0065610.s001.tif]
